# Low-Temperature 3D Printing Technology of Poly (Vinyl Alcohol) Matrix Conductive Hydrogel Sensors with Diversified Path Structures and Good Electric Sensing Properties

**DOI:** 10.3390/s23198063

**Published:** 2023-09-24

**Authors:** Qian Zhao, Chang Liu, Yanjiao Chang, Han Wu, Yihao Hou, Siyang Wu, Mingzhuo Guo

**Affiliations:** 1 The Key Laboratory of Bionic Engineering, Ministry of Education, Jilin University, Changchun 130025, China; 2 College of Food Science and Engineering, Jilin University, Changchun 130062, China

**Keywords:** hydrogel sensor, low-temperature 3D printing, conductivity, structure design, high sensitivity

## Abstract

Novel and practical low-temperature 3D printing technology composed of a low-temperature 3D printing machine and optimized low-temperature 3D printing parameters was successfully developed. Under a low-temperature environment of 0–−20 °C, poly (vinyl alcohol) (PVA) matrix hydrogels including PVA-sodium lignosulphonate (PVA-LS) hydrogel and PVA-sodium carboxymethylcellulose (PVA-CMC) hydrogel exhibited specific low-temperature rheology properties, building theoretical low-temperature 3D printable bases. The self-made low-temperature 3D printing machine realized a machinery foundation for low-temperature 3D printing technology. Combined with ancillary path and strut members, simple and complicated structures were constructed with high precision. Based on self-compiling G-codes of path structures, layered variable-angle structures with high structure strength were also realized. After low-temperature 3D printing of path structures, excellent electrical sensing functions can be constructed on PVA matrix hydrogel surfaces via monoplasmatic silver particles which can be obtained from reduced reactions. Under the premise of maintaining original material function attributes, low-temperature 3D printing technology realized functionalization of path structures. Based on “3D printing first and then functionalization” logic, low-temperature 3D printing technology innovatively combined structure–strength design, 3D printable ability and electrical sensing functions of PVA matrix hydrogels.

## 1. Introduction

As a kind of water-soluble synthetic polymer, Poly (vinyl alcohol) (PVA) has the perfect biodegradability, biocompatibility, nontoxicity and large number of hydroxyl units [1,2,3], which provides sufficient mechanical strength and flexibility base for preparation of PVA matrix hydrogels. Combined with inorganic fillers including lignin, graphene oxide, nano clay, ammonium sulfate ions and so on [4,5,6,7], PVA hydrogels exhibit “green” application features in drug delivery systems, wound dressings and especially soft sensors [8,9,10]. For example, Zhang and co-workers prepared a kind of PVA hydrogel with relatively high mechanical strength and conductivity on the raw material base of biomass sodium lignosulfonate and PVA [11]. By physically mixing the PVA and glycerol in water as the gel matrix, followed by soaking in a saturated NaCl aqueous solution, the PVA/glycerol/NaCl ionic hydrogel sensors exhibited excellent transparency, stretchability, mechanical strength, toughness and excellent stretching sensitivity with a gauge factor of 4.01 [12]. Even though PVA hydrogels have impressive advantages, the simple sample structures are always shapes of strip and chunk. Therefore, with the increasing extensive application of PVA hydrogels, complex and diversified structure patterns are important application restrictions and bottlenecks.

3D printing technology has the advantages of material universality, convenience, cleanliness and environmental protection and continuous production efficiency [13,14,15,16], and is widely used in polymer material preparation and is treated as an effective method for resolving the restrictions and bottlenecks of PVA hydrogels. The existing typical 3D printing technologies are material jetting and material extrusion (fused deposition modelling and direct ink writing) [17,18,19,20,21,22]. As the most common 3D printing pattern, the material extrusion 3D printing technology exhibits wide material adaptability including polymers, metals, ceramics and so on [23]. In practical application, direct-ink-writing 3D printing can be easily modified to combine temperature control systems and other auxiliary modules inexpensively, which is treated as the main pattern of the laboratory-built 3D printing machine. In order to realize the complex and diversified structural patterns of PVA hydrogels via direct-ink-writing 3D printing, the rheology characteristics of PVA hydrogel ink are the key points to resolve. The common method for realizing the printable ability of hydrogels is the addition of accessory ingredients [24,25,26], which adjusts the viscosity, storage modulus and loss modulus of hydrogel inks effectively. But, the incidental property variations including strength, toughness and crosslinking density change the original material characteristics and application demands. Namely, how to realize the printable ability of PVA hydrogel and maintain the original or specific material properties are the inevitable key points and challenges. Based on the combination of material properties and printable abilities, a new novel 3D printing technology is the innovative solution.

The polymerization reaction of PVA hydrogels via freeze–thaw cycles in our previous study provided the breakthrough point of 3D printing feasibility for authors to combine material properties and printable abilities. In our previous study [27], a kind of PVA matrix conductive hydrogel with excellent conductivity and sensitivity was prepared via freeze–thaw cycles and an in situ reduction reaction of silver particles. During the polymerization of PVA and sodium lignosulphonate (LS) under an environment of low temperature of −18 °C for 12 h, a PVA-LS hydrogel reaction liquid changed from a liquid state to the final gel state. Namely, the viscosity value of the PVA-LS hydrogel increased gradually in polymerization, which disclosed the important effect of temperature on the rheology characteristics of the PVA-LS hydrogel. There must be a specific temperature range in the freeze–thaw cycles, leading to the appropriate rheology characteristic for 3D printing. Inspired by characteristics of the low-temperature polymerization process of PVA hydrogel, the authors decided to innovate a new 3D printing technology which can realize effective diversified and complex structures of PVA hydrogels and maintain the original or specific material properties. The convenient, high-efficiency and feasible low-temperature 3D printing machine which composed of the direct-ink-writing 3D printer and a low-temperature control system is the foundation of 3D printing of PVA hydrogels.

In this study, the low-temperature 3D printing machine and corresponding 3D printing parameters for PVA matrix hydrogels were assembled and developed, respectively. The low-temperature 3D printing machine can be divided into four parts including a parallel-shaft drive mechanism, a hydraulic extrusion mechanism, a low-temperature control device and an electric control device. The parallel-shaft drive mechanism and hydraulic extrusion mechanism constituted the direct-ink-writing device of the low-temperature 3D printing machine, which realized steady and continuously three-dimensional movements of the nozzle and controllable extrusion of the PVA hydrogel reaction liquid. In the low-temperature environment ranging from 0 °C to −20 °C, PVA matrix hydrogels realized high-precision 3D printing. Using the self-made low-temperature 3D printing machine, PVA-LS and PVA-sodium carboxymethylcellulose (CMC) hydrogels with complex and diversified structures were 3D printed. Considering the precondition of maintaining original mechanical strength and reduction reaction matrix role, the innovative low-temperature 3D printing technologies of PVA matrix hydrogels provided a simple and highly effective method for overcoming the application bottleneck of PVA matrix hydrogels.

## 2. Experimental Section

### 2.1. Materials

PVA (degree of hydrolysis 99%, M_w_ ≈ 130,000, [C_2_H_4_O]_n_) was obtained from Sigma-Aldrich (Shanghai) Trading Co., Ltd., Shanghai, China. LS (M_w_ ≈ 534.51) was purchased from Hefei BASF Biotechnology Co., Ltd., Hefei, China. CMC (M_w_ ≈ 534.51) was acquired from Aladdin Industrial Inc., Shanghai, China. Dimethyl sulfoxide (DMSO), silver nitrate (AgNO_3_), K30 polyvinylpyrrolidone (PVP) and ascorbic acid (VC, C_6_H_8_O_6_) were obtained from Tianjin Fuyu Fine Chemical Co., Ltd., Tianjin, China; Tianjin Beilian Fine Chemicals Development Co., Ltd., Tianjin, China; Xi’an Yuelai Pharmaceutical Excipients Co., Ltd., Xi’an, China. and Tianjin Guangfu Technology Development Co., Ltd., Tianjin, China, respectively. All the reagents were used without pretreatment.

### 2.2. Design and Components of Low-Temperature 3D Printing Machine

The low-temperature 3D printing machine is composed of a parallel-shaft drive mechanism, a hydraulic extrusion mechanism, a low-temperature control device and an electric control device. In order to realize the application of objects of small-space–occupancy ratios, stable and efficient operation, continuous movement of the nozzle, 3D printing precision and controllable extrusion of hydrogel inks, the main load bearing structures are aluminium profiles (Model 4040F). Under the conditions of satisfying operating requirements and reducing manufacturing costs, the other load bearing structures were 3D printed high-strength polylactic resin (Sanweicube Co., Ltd., Shenzhen, China). The components of the low-temperature 3D printing machine are listed in Table 1.

The integral structure of the low-temperature 3D printing machine is a secure trigonal prism. The three-dimensional location of the nozzle is realized by three independent pulleys which were vertically installed on aluminium profiles. Under the control of programs, the three pulleys operate synergistically via stepping motors and synchronous belts, which drive the three-dimensional movements of nozzle. The nozzle connects with an ink storage tube. In order to ensure 3D printing precision and reduce the effect of inertance on the movement precision of the ink storage tube, the distal extrusion pattern is adopted. Driven force of the hydraulic extrusion mechanism results from stepping motors and synchronous belts. The pressure medium is water. The hydraulic extrusion mechanism is located on the top of trigonal prism structure, which connects with the ink storage tube via a plastic hose. The low-temperature control device and electric control device are located on the bottom of the trigonal prism structure, which constructs a low-temperature environment and drives the stepping motors, respectively. The length, width and height of the low-temperature 3D printing machine are 300 mm, 300 mm and 800 mm, respectively. The movement speed range of the nozzle is 10–60 mm/s. The extrusion speed range is 0.2–1.2 mm/s. The low-temperature range is 0–−20 °C.

### 2.3. Low-Temperature 3D Printing of PVA Matrix Hydrogels

#### 2.3.1. Preparation of PVA Matrix Hydrogel Reaction Liquids

In order to verify the feasibility of the low-temperature 3D printing technology and the effectiveness of the low-temperature 3D printing machine, two kinds of PVA matrix hydrogels including PVA-LS hydrogel and PVA-CMC hydrogel were prepared.

The preparation processes of the PVA-LS hydrogel were as follows: 45 mL of a co-solvent mixture of DMSO and deionized water with a volume of 4:1 was prepared via magnetic stirring. LS weighing 0.15 g was added into the co-solvent mixture of DMSO and deionized water and stirred and sonicated for 10 min and 20 min at room temperature, respectively. Then 4.85 g of PVA was added into the mixed solution and mechanically stirred at 400 rpm for 2 h at 140 °C. When the temperature cooled to 60 °C, the final PVA-LS mixed solution was slowly poured into the ink storage tube for low-temperature 3D printing.

The preparation processes of the PVA-CMC hydrogel were as follows: 0.15 g CMC was placed on the bottom of a beaker and adequately infiltrated by ethanol for 2 min. Then, 45 mL deionized water was poured into the beaker and sonicated for 20 min. Then, 4.85 g PVA was added into the CMC solution and continuously magnetically stirred at 800 rpm to obtain a homodisperse PVA-CMC mixed solution. Then, the PVA-CMC mixed solution was stirred at 400 rpm for 2.5 h at 95 °C. When the temperature cooled to 60 °C, the final PVA-CMC mixed solution was slowly poured into the ink storage tube for low-temperature 3D printing.

#### 2.3.2. Low-Temperature 3D Printing Process of PVA Matrix Hydrogels

Before low-temperature 3D printing, a series of structural models and corresponding STL files were constructed via Solidworks. RepetierHost was used to connect the low-temperature 3D printing machine and to control the 3D printing process. After steady installation of the ink storage tube with the PVA matrix hydrogel ink, the low-temperature control device was started, firstly to obtain a low-temperature environment of −20 °C. Then, the hydraulic extrusion mechanism was preloaded to ensure continuous extrusion of the PVA hydrogel ink in the 3D printing process. Under the control of the electric control device, the parallel-shaft drive mechanism and the hydraulic extrusion mechanism jointly prepared the structural models. After low-temperature 3D printing, 3D printed samples were placed in a −20 °C environment for 12 h and then thawed at room temperature for 2 h. The polymerized PVA-LS and PVA-CMC hydrogels were immersed in deionized water for 4 days (changing water every 12 h) to remove the DMSO completely.

#### 2.3.3. Functionalization of Low-Temperature 3D Printing PVA Matrix Hydrogels

In order to verify the innovativeness of the low-temperature 3D printing technology which maintained the original functionalization properties of PVA-LS and PVA-CMC hydrogels, a silver particles reduction reaction was conducted. After mixing AgNO_3_, VC and PVP solution with specific concentrations, PVA-LS and PVA-CMC hydrogels were soaked in the mixed AgNO_3_-PVP solution for 8 h and then soaked in the mixed VC-PVP solution for 24 h. After washing with deionized water, the low-temperature 3D printed conductive PVA matrix hydrogels were prepared. In order to obtain optimal conductivity and sensitivity, the parameters of the silver particles reduction reaction were obtained via our previous study. The concentration of AgNO_3_ and VC were 1 M and 0.08 M, respectively. The mass fraction of PVP was 10 wt.%.

### 2.4. Material Characteristics

#### 2.4.1. Rheology Tests

A rotational rheometer (DHR, TA Instruments, New Castle, DE, USA) with a parallel plate geometry of 40 mm in diameter and a gap of 0.55 mm was employed to analyse the low-temperature rheology properties of the PVA matrix hydrogel reaction liquids. The strain sweep range was 0.1–1000%. Under the condition of the oscillation mode, the frequency was 1 Hz. Shear rate was 6.28 rad/s. In a temperature variation range of 25–−20 °C, viscosity, storage modulus and loss modulus were analysed.

#### 2.4.2. Mechanical Tests

The universal testing machine with a constant loading rate of 100 mm·min^−1^ was used to obtain the tensile strength of the low-temperature 3D printed PVA matrix hydrogels. The sample size was 40 mm × 5 mm × 4 mm (Length × Width × Height). Average values of stress and strain were calculated from three individual measurements.

#### 2.4.3. Microstructure and Phase Component Tests

In order to observe the microstructure and distribution pattern of the reduced silver particles, the cross-sections of the PVA-LS and PVA-CMC hydrogels after the silver reduction reaction were observed by field-emission scanning electron microscopy (SEM, XL-30, FEI Company) equipped with an energy-dispersive spectrometer. All samples were cryofractured in liquid nitrogen first and then freeze-dried for 48 h via a freeze-drying oven (LGJ-10C, Beijing Four Ring Scientific Instrument Factory Co., Ltd., Beijing, China).

In order to verify the phase component of the silver particles on the PVA matrix hydrogel surfaces, X-ray diffraction (XRD, MAXima_X XRD-7000) with Cu Kα radiation in the range of 10–90° was conducted. The scan speed was 4 deg/min. The freeze-dried PVA matrix hydrogels with silver particles were pressed into sheets for XRD analysis.

#### 2.4.4. Conductivity and Electrical Sensing Tests

The conductivity of the low-temperature 3D printing PVA matrix hydrogels was measured via a digital-display DC power supply. The digital-display DC power supply exhibited the voltage (U) and current (I) loaded on the samples in real time, which provided resistance (R) calculation data via Ohm’s law of R = U/I. The parallel-connected “JLU” LED circuit board was used to exhibit conductivity intuitively.

The gauge factor (GF) was also calculated and analysed. The PVA matrix hydrogels with silver particles were fixed on the universal testing machine with a constant stretching rate of 10 mm/min. A digital-display multimeter (KEYSIGHT, 34465A) with an NPLC value of 0.02 was used to record resistance variation in the stretching process. The relative change in the amount of resistance (ΔR) was calculated as follows:

ΔR = R − R_0_ (1)

where R and R_0_ represented the real-time resistance and initial resistance, respectively, which can be obtained via the digital-display multimeter.

The GF was calculated as follows:

(2) $$\mathrm{GF}=\frac{\Delta R}{\varepsilon R_{0}}\mathrm{GF}=\frac{\Delta R}{\varepsilon R_{0}}$$

where *ε* was the specific strain of the PVA matrix hydrogels in the stretching process.

## 3. Results and Discussion

### 3.1. Low-Temperature Rheology Analysis of PVA Matrix Hydrogels

**Figure 1:**
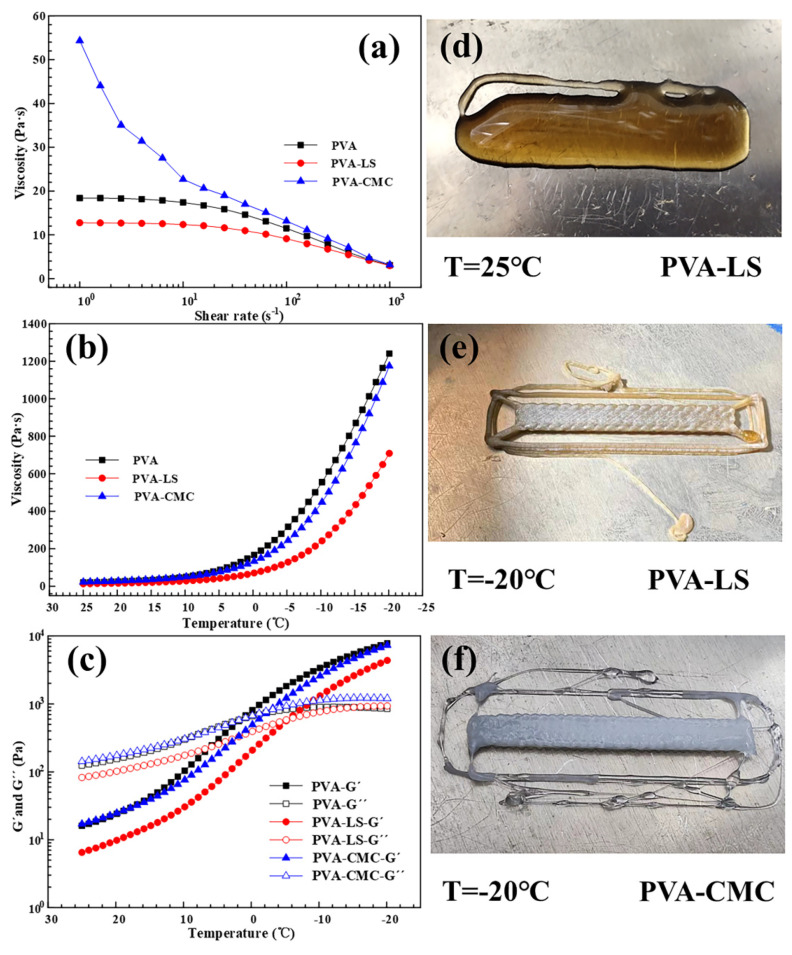
Low-temperature rheology characteristics including (**a**) viscosity at 25 °C, (**b**) viscosity variation ranging from 25 °C to −20 °C, (**c**) storage modulus G′ and loss modulus G″, (**d**) PVA-LS pattern at 25 °C, (**e**) PVA-LS pattern at −20 °C and (**f**) PVA-CMC pattern at −20 °C.

Low-temperature rheology characteristics of the PVA matrix hydrogels are the theoretical basis of the low-temperature 3D printing technology and mechanical framework. Figure 1a shows viscosity variation of PVA-LS and PVA-CMC hydrogel reaction liquids and pure PVA liquid. With the increase of shear rate, viscosity values of the PVA matrix hydrogels decreased gradually, exhibiting a shear-thinning phenomenon. With the further increase of shear rate, the entanglements of the macromolecular segments were straightened, leading to the viscosity values of the PVA matrix hydrogels decreasing linearly. The PVA matrix hydrogel reaction liquids exhibited the typical Newtonian fluid phenomenon at 25 °C. During the temperature declining process from 25 °C to −20 °C, viscosity values of the PVA-LS, PVA-CMC and pure PVA increased gradually, as shown in Figure 1b. Compared with Figure 1a, viscosity of the PVA matrix hydrogel reaction liquids had obvious increases. The addition of LS and CMC maintained the original viscosity characteristic of PVA. Based on viscosity characteristics, the low-temperature environment enabled the primary feasibility of the low-temperature 3D printing. In order to investigate the theoretical 3D printable ability, the storage modulus and loss modulus of the PVA matrix hydrogel reaction liquids were tested, as shown in Figure 1c. The crossover point of the storage modulus and loss modulus was treated as an indication value of the breakdown of the gel network structure and the transition of the quasi-liquid phase. PVA and PVA matrix hydrogels all had crossover points as shown in Figure 1c. The storage modulus and loss modulus curves of the PVA-LS and PVA-CMC hydrogel reaction liquids intersected in a temperature range between −10 °C and 0 °C. When the temperature was lower than −10 °C, the storage modulus was higher than the loss modulus. PVA-LS exhibited elastic deformation and a solid state. When the temperature was higher than −5 °C, the storage modulus was lower than the loss modulus. PVA-LS exhibited viscous deformation and a liquid state. A similar variation phenomena between storage modulus and loss modulus also existed in PVA-CMC and PVA. As shown in Figure 1c, the viscosity increased suddenly, and fluidity decreased, which existed in PVA matrix hydrogels. Based on the influence of the low-temperature environment, the PVA matrix hydrogel reaction solutions had perfect rheology properties which can store energy via elastic deformations and lose energy via viscous deformations. The addition of LS and CMC maintained the original modulus characteristics of PVA. As shown in Figure 1b,c, the PVA matrix hydrogel reaction solutions exhibited specific low-temperature rheology properties, which adequately proved the feasibility of the low-temperature 3D printing technology. Figure 1d–f exhibits the 3D printed sample patterns of the PVA matrix hydrogels at 25 °C and −20 °C, respectively. The failed 3D printing result shown in Figure 1d at 25 °C can be found in the supplemental movie (Supporting Information M1). The low-temperature of −20 °C realized the controllable extrusion and perfect structure retention properties of PVA matrix hydrogels effectively, which intuitively proved the effectiveness of the theoretical analysis of low-temperature rheology.

### 3.2. Construct and Operation of Low-Temperature 3D Printing Machine

**Figure 2:**
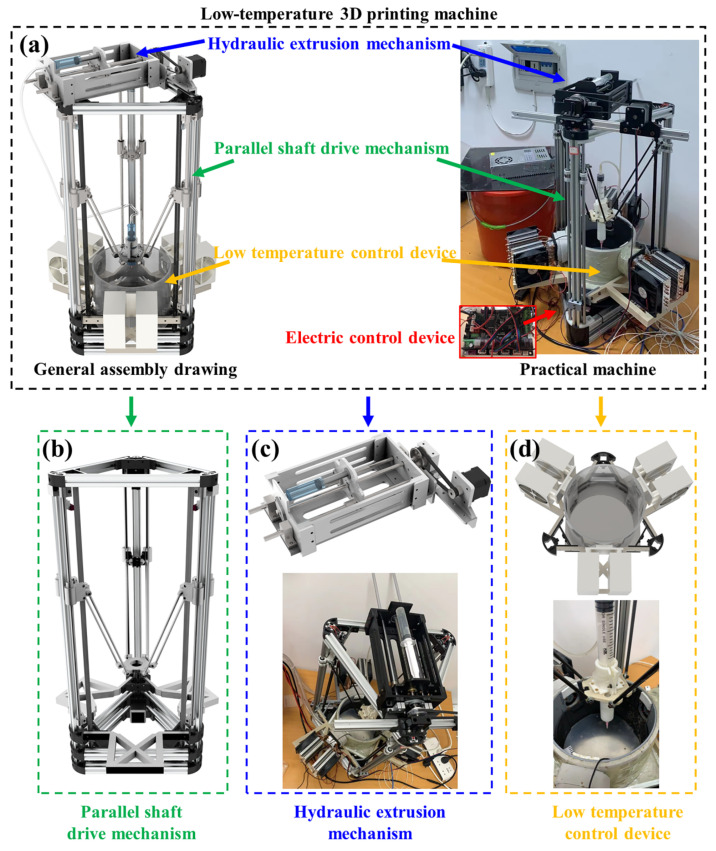
The (**a**) general assembly drawing and corresponding assembly drawings of (**b**) parallel-shaft drive mechanism, (**c**) hydraulic extrusion mechanism and (**d**) low-temperature control device of the low-temperature 3D printing machine.

In order to realize the 3D printing technology of PVA matrix hydrogel reaction liquids, a self-made low-temperature 3D printing machine was designed and constructed. Figure 2a exhibits the general assembly drawing and practical pattern of the self-made low-temperature 3D printing machine. The low-temperature 3D printing machine was designed with a triangular prism structure to obtain high structural stability, which was composed of a hydraulic extrusion mechanism, a parallel-shaft drive mechanism, a low-temperature control device and an electric control device. The parallel-shaft drive mechanism was the main body frame of the low-temperature 3D printing machine. Three independent pulleys on the three vertical aluminium profiles drove movement of the nozzle on the X axis, Y axis and Z axis via the connection of parallel shafts. Pulleys were driven by stepping motors. Accurate and rapid positioning properties were advantages of the parallel-shaft drive mechanism. The hydraulic extrusion mechanism undertook the extrusion of the PVA matrix hydrogel reaction liquids, which were located on the top of the parallel-shaft drive mechanism. The distalis extrusion pattern reduced motion inertia of the storage tube and maintained 3D printing precision. The stepping motor and water were utilized as the driving source and pressure medium, respectively, controllably extruding the PVA matrix hydrogel reaction liquid in the storage tube. The low-temperature control device realized a low-temperature environment ranging from 0 °C to −20 °C via a semiconductor chilling plate. The low-temperature control device was controlled by an individual power supply. Circulating water was used for heat dissipation of the semiconductor chilling plate and was stored in the red bucket. The electric control device located on the bottom of the parallel-shaft drive mechanism controlled the synergistic operation of the hydraulic extrusion mechanism and the parallel-shaft drive mechanism. The operation process of the complete machine can be found in the supplemental movie (Supporting Information M2). The steady and accurate operation process proved the feasibility of the low-temperature 3D printing machine.

Figure 2b shows the assembly drawings of the parallel-shaft drive mechanism. The parallel shaft was composed of three groups of connecting rods. One group of connecting rods was composed of two parallel fish-eye rods. The parallel shaft structure had high load capacity. One side of the fish-eye rod connected with a pulley, the other side connected with a triangular platform which was used for installing the storage tube. The hybrid stepping motors with high output torque and positional accuracy were fixed on aluminium profiles. A hybrid stepping motor, type 42BYG60, was utilized as the drive motor of the parallel-shaft drive mechanism. The corresponding detailed parameters are listed in Table 2.

In order to maintain high positional accuracy and sufficient drive bite force of the parallel-shaft drive mechanism, a synchronizing wheel, type 2GT20, with 20 teeth and 2 mm teeth space was used. The type of synchronous belt was a 2GT with a pitch line of 300 mm. The synchronizing wheel and synchronous belt system connected with a jackscrew. The polish rods with a length of 520 mm and a diameter of 8 mm were treated as guide rails of the pulleys to enhance positional accuracy of the parallel-shaft drive mechanism. The aluminium profiles were frame materials for the low-temperature 3D printing machine, which provided sufficient strength and space for arrangement of the mechanical structure and wiring harness. Considering the space requirements of 3D printing, aluminium profiles with lengths of 300 mm and 600 mm were used for construction of the X axis, and the Y axis and Z axis, respectively. The frame structures which were used to install the cooling fans were 3D printed with polylactic acid.

Figure 2c exhibits the assembly drawings of the hydraulic extrusion mechanism, which was also driven via a synchronizing wheel and synchronous belt system with a deceleration transmission ratio of 1:3. The small synchronizing wheel connected with the hybrid stepping motor, type 42BYG60, via a jackscrew, driving the big synchronizing wheel via the synchronous belt. The big synchronizing wheel connected with a screw rod via a jackscrew. The rotating screw rod led to the movement of the sliding block, pushing water out from the injector. The polish rods with a length of 300 mm and diameter of 8 mm were treated as guide rails of the sliding block to enhance positional accuracy. The extruded water was squeezed into another empty injector, pushing the piston core rod of the injector out of the work drum. The empty injector connected with the storage tube via a triangular platform. The extruded piston core rod pushed the PVA matrix hydrogel reaction liquid out of the storage tube. Combined with the calculation and debugging, the feed speed of the hydraulic extrusion mechanism matched with the movement speed of the triangular platform in the parallel-shaft drive mechanism. Besides the metalline components and parts, the other structural components were also 3D printed with polylactic acid to enhance packaging efficiency.

Figure 2d shows the assembly drawings of the low-temperature control device, which can construct a low-temperature environment of −20 °C. When the PVA matrix hydrogel reaction liquid was extruded by the hydraulic extrusion mechanism, the low-temperature control device provided a controllable and steady low-temperature environment for 3D printing. The self-made low-temperature 3D printing machine combined advantages of a direct-ink-writing 3D printing machine and a low-temperature system, which constructed a steady and effective platform to realize the low-temperature rheology properties of PVA matrix hydrogels.

### 3.3. Structure Properties of Low-Temperature 3D Printing Technology

#### 3.3.1. Low-Temperature 3D Printing of Simple Structures

**Figure 3:**
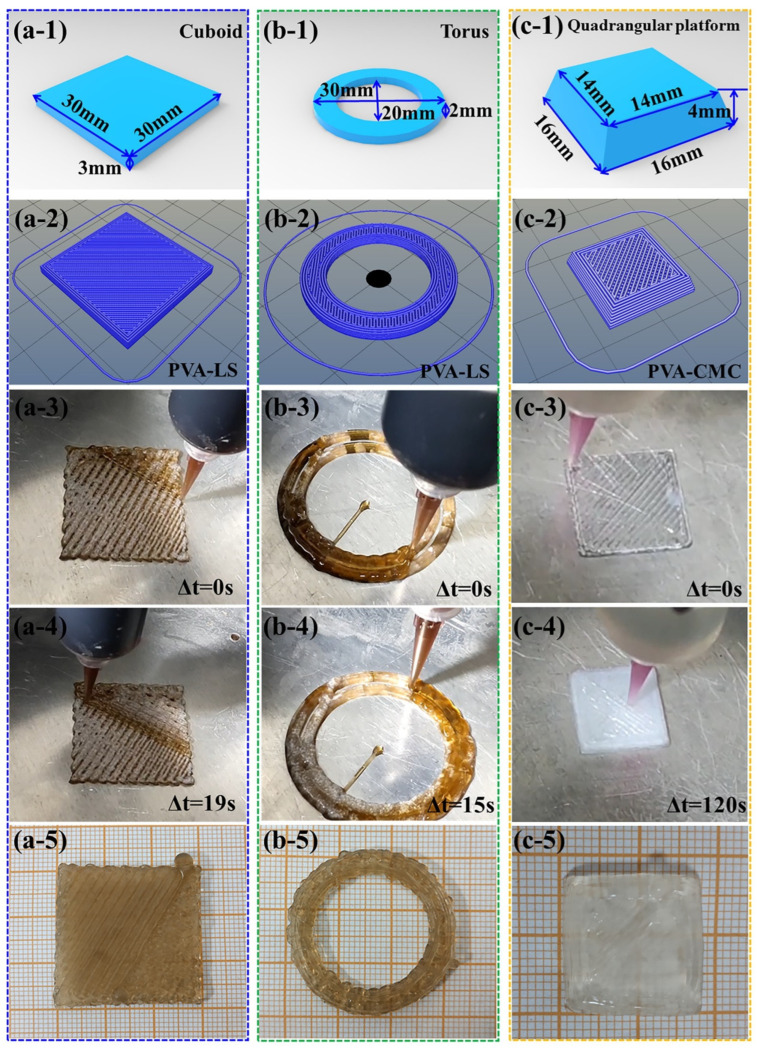
Low-temperature 3D printing process of PVA matrix hydrogels with (**a-1**)–(**a-5**) cuboid structure, (**b-1**)–(**b-5**) torus structure and (**c-1**)–(**c-5**) quadrangular platform structure.

**Figure 4:**
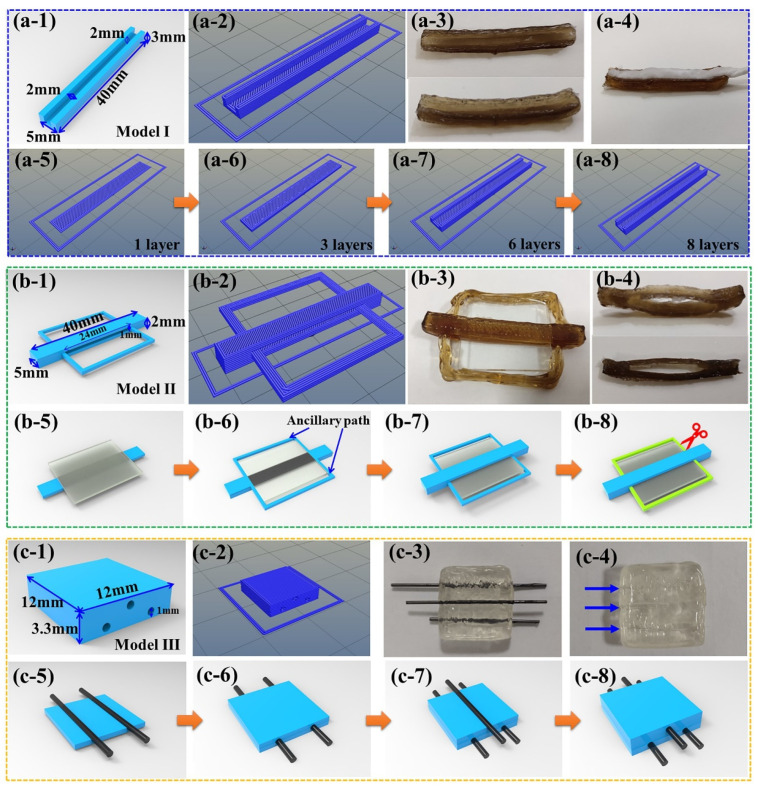
Low-temperature 3D printing process of PVA matrix hydrogels with complex structures of (**a-1**)–(**a-8**) Model I, (**b-1**)–(**b-8**) Model II and (**c-1**)–(**c-8**) Model III.

**Figure 5:**
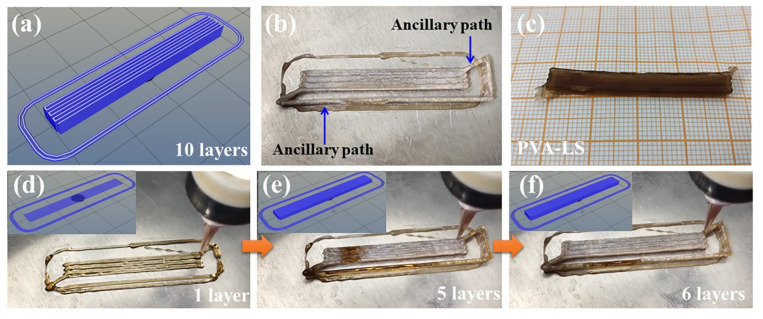
(**a**) self-compiling G-code pattern, (**b**) practical pattern, (**c**) final pattern and low-temperature 3D printing process of (**d**) 1 layer, (**e**) 5 layers and (**f**) 6 layers of the parallel path structure.

A series of typical common structures were designed to verify the operational effectiveness of the low-temperature 3D printing machine. Figure 3(a-1–a-5) depicts the process and material object of PVA-LS hydrogels with a cuboid structure. Based on a large number of debugging experiments, the detailed low-temperature 3D printing parameters which can realize a practical 100% filling rate are listed in Table 3.

The size of the cuboid structure was 30 mm × 30 mm × 3 mm (Length × Width × Height), as shown in Figure 3(a-1). Figure 3(a-2) exhibits the 3D printing slice path pattern of a cuboid structure with eight layers. The detailed low-temperature 3D printing process of the cuboid structure can be observed in the supplemental movie (Supporting Information M3). In order to be convenient for introducing a low-temperature 3D printing process, an initial time of Δt = 0 s was artificially set in Figure 3(a-3). At the point of Δt = 0, the nozzle which linked with the storage tube was at the top-right corner of the 3D printed sample. Several extrusive PVA-LS paths with the original colour of the PVA-LS hydrogel reaction liquid maintained the initial path structure without collapsing. Moreover, the extrusive path structure was not solidified. After 19 s, the nozzle moved to the middle part of the 3D printed sample in Figure 3(a-4). The extrusive continuous path structure without collapse also exhibited the original colour of the PVA-LS hydrogel reaction liquid. But, the colour of the 3D printed paths in the top-right corner were white, which indicated the corresponding path structure was solidified. When the designed paths depicted in Figure 3(a-2) were 3D printed one by one, the low-temperature 3D printing process of the cuboid structure was finished. After sufficient cycles of freezing and thawing, the PVA-LS hydrogel cuboid was finished, as shown in Figure 3(a-5). Compared with the designed size, the size of the final PVA-LS hydrogel cuboid was 30 mm × 30 mm × 3 mm (Length × Width × Height), which proved the high 3D printing precision of the self-made low-temperature 3D printing machine.

In order to verify the repeatability of the low-temperature 3D printing machine, a torus structure was designed. The corresponding dimension parameters are exhibited in Figure 3(b-1). The 3D printing path structure with five layers is shown in Figure 3(b-2). The practical low-temperature 3D printing process can be found in the supplemental movies (Supporting Information M4). The time which described the nozzle position in Figure 3(b-3) was also defied as Δt = 0 s. The nozzle was on the second layer of the torus structure. In the left bottom part, the 3D printing path maintained the designed unbroken path structure. After the continuous and unhindered low-temperature 3D printing process, the nozzle moved to top right corner. As shown in Figure 3(b-4), the left bottom part depicted in Figure 3(b-3) was solidified and maintained the original 3D printed structure perfectly, which provided a solid structural base for the third layer. After sufficient cycles of freezing and thawing, the practical low-temperature 3D printed torus sample had high dimensional accuracy via comparison between Figure 3(b-1,b-5).

The low-temperature 3D printing of the cuboid and torus structures effectively verified the feasibility of low-temperature 3D printing. The quadrangular platform structure depicted in Figure 3(c-1) was designed to investigate the accumulation property of the PVA-CMC hydrogel. The slice path structure with seven layers is exhibited in Figure 3(c-2). The detailed low-temperature 3D printing process can be found in the supplemental movies (Supporting Information M5). Attributed to the low-temperature rheology properties, PVA-CMC reaction liquid can also realize continuous and steady low-temperature 3D printing, which was similar to the PVA-LS reaction liquid. The first layer was 3D printed and maintained the original designed path structure without collapse, as shown in Figure 3(c-3). After 120 s, the fourth layer was low-temperature 3D printing on the solidified first three layers as depicted in Figure 3(c-4). After accumulation layer by layer, the final low-temperature 3D printed PVA-CMC quadrangular platform structure was complete, as exhibited in Figure 3(c-5), proving the high structure accumulation and precision properties of the low-temperature 3D printing machine.

The low-temperature 3D printing process of simple structures depicted in Figure 3 indicated the operational effectiveness of the self-made low-temperature 3D printing machine. Based on the low-temperature rheology characteristics of PVA matrix hydrogels, the low-temperature 3D printing technologies including the steady low-temperature 3D printing machine and controllable parameters provided preparation technology bases for PVA matrix hydrogels with complicated structures.

#### 3.3.2. Low-Temperature 3D Printing of Complex Structures

**Figure 6:**
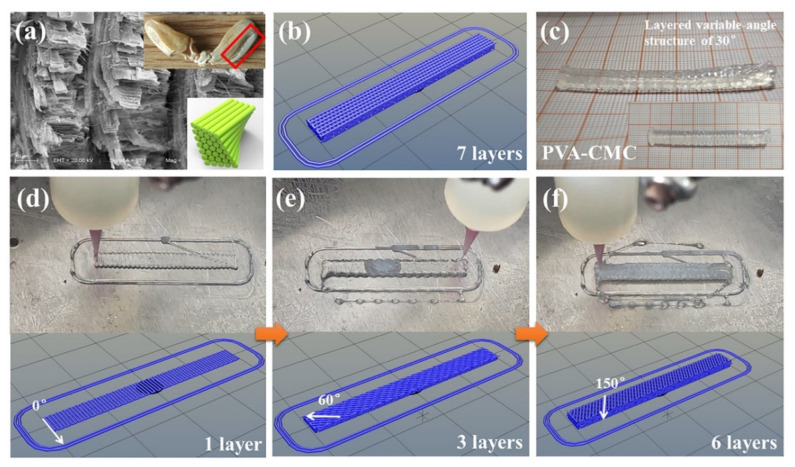
(**a**) microstructure of spearer propodus of mantis shrimp and (**b**) self-compiling G-code pattern, (**c**) final pattern and low-temperature 3D printing process of (**d**) 1 layer, (**e**) 3 layers and (**f**) 6 layers of layered variable-angle structure.

**Figure 7:**
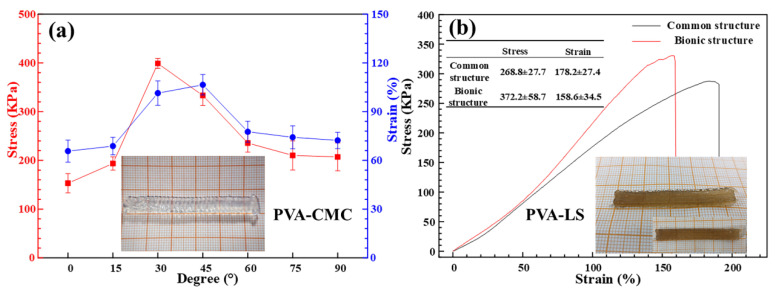
Mechanical strength analysis of low-temperature 3D printing (**a**) PVA-CMC and (**b**) PVA-LS hydrogels via self-compiling G-code.

**Figure 8:**
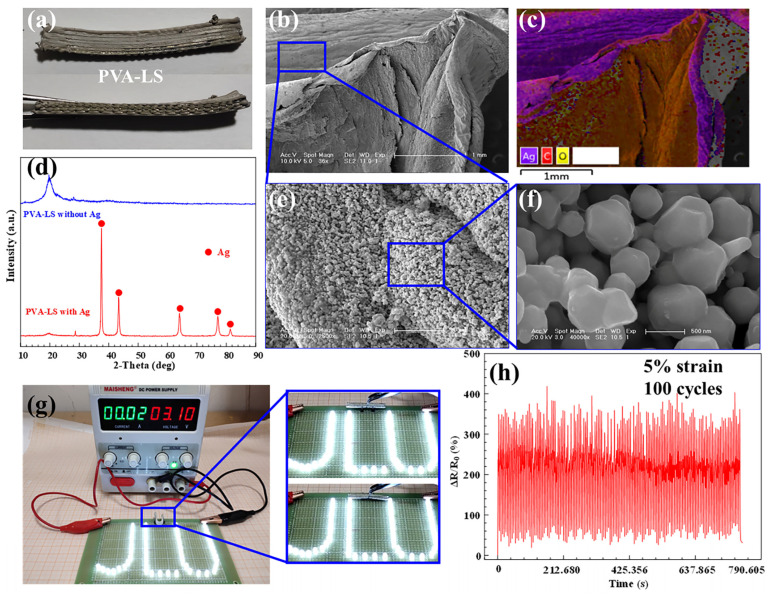
(**a**) material object, (**b**) morphology, (**c**) energy spectrum, (**d**) XRD curves, (**e**) microstructure and (**f**) amplifying morphology of Ag nano particles, (**g**) electrical conductivity and (**h**) ΔR/R_0_ with 5% strain for 100 cycles of low-temperature 3D printing PVA-LS conductive hydrogels.

After the constructions of simple structures as shown in Figure 4, the complex structure construction property with strut member was analysed, as shown in Figure 4. The low-temperature 3D printable ability of undersized and exquisite structures was the steady base of complex structure construction with strut member of PVA-LS and PVA-CMC. Therefore, the Model I of a groove cuboid with detailed dimensions as shown in Figure 4(a-1) was adopted. The depth and width of the groove were 2 mm. The width of the prominent part was only 1.5 mm. As shown in Figure 4(a-2), the path structure with nine layers was constructed via the slicing software of CuraEngine. Combined with the continuous extrusion, the low-temperature 3D printing machine realized the path structure and 3D printed the groove cuboid successfully, which can be seen in Figure 4(a-3). The degreasing cotton in the 3D printed groove directly exhibited the undersized structure construction property of low-temperature technology. Combined with the practical low-temperature 3D printing process shown in the supplemental movie (Supporting Information M6), the 3D printing process of the path structure is listed in Figure 4(a-5–a-8). The solid cuboid with dimensions of 40 mm × 5 mm × 1 mm (Length × Width × Height) was divided into three layers. Under the positive role of low-temperature rheology, the first three layers, with 100% filling rate, were 3D printed layer by layer. On the base of the solidified first three layers, the other six layers of the two prominent parts with height of 2 mm were low-temperature 3D printed successfully.

In order to verify the low-temperature 3D printing feasibility of complex structures via strut members, Model II of the cuboid with a thru hole was designed. A glass sheet with dimensions of 24 mm × 20 mm × 1 mm (Length × Width × Height) was treated as a strut member. Attributed to the continuity of the low-temperature 3D printing process, ancillary paths were adopted to reserve enough operation time for placing the glass strut member. The detailed shape and dimensions of Model II are exhibited in Figure 4(b-1). The thru hole dimensions of the cuboid were 24 mm × 5 mm × 1 mm (Length × Width × Height). The 3D printing path structure with nine layers obtained from the slicing software of CuraEngine is shown in Figure 4(b-2). Figure 4(b-3) shows the low-temperature 3D printed cuboid with the glass sheet strut member. Figure 4(b-4) shows the final shape of Figure 4(b-3) without ancillary paths, which proved the feasibility of the structural design of Model II. The supplemental movie (Supporting Information M7) exhibits the detailed low-temperature 3D printing process of Model II. Figure 4(b-5–b-8) shows the schematic diagrams of the main procedures of the low-temperature 3D printing process of Model II. The first three layers with dimensions of 40 mm × 5 mm × 0.5 mm (Length × Width × Height) were low-temperature 3D printed, serving as the base of the thru hole structure. When the fourth layer and corresponding ancillary path started, the glass strut member was placed on the solidified PVA-LS hydrogel reaction liquid. Then, the other three layers were 3D printed on the solidified PVA-LS hydrogel reaction liquid and glass strut member shown in Figure 4(b-1). After sufficient cycles of freezing and thawing, the glass strut member and low-temperature 3D printed ancillary paths were removed and cut off, respectively, which resulted in the final low-temperature 3D printed PVA-LS cuboid with thru hole shown in Figure 4(b-4).

In order to verify whether the low-temperature 3D printing technology can realize complicated structures with multiple strut members or not, PVA-CMC hydrogel reaction liquid was used for the low-temperature 3D printing of Model III. As shown in Figure 4(c-1), Model III was a cube with three thru holes. Figure 4(c-2) exhibits the path structure with the nine layers of Model III. The size dimensions of the columniform thru hole was Φ1 mm × 12 mm (Diameter × Length). Figure 4(c-3,c-4) exhibits the practical low-temperature 3D printed cubic with and without strut members, respectively. The supplemental movie (Supporting Information M8) exhibits the detailed low-temperature 3D printing process of Model III. Figure 4(c-5–c-8) exhibits the main low-temperature 3D printing processes of Model III. After the solidification of the first three layers, two columniform strut members were placed in sequence. Then, the other four layers were low-temperature 3D printed on the strut members. Another columniform strut member was also placed on the solidified PVA-CMC hydrogel reaction liquid. After the low-temperature 3D printing of the rest of the path structures, Model III was constructed. After sufficient cycles of freezing and thawing, the low-temperature 3D printed PVA-CMC cube with three thru holes shown in Figure 4(c-1) was successfully obtained. Combined with the low-temperature 3D printing process of PVA matrix hydrogels with Model I, Model II and Model III, the novel low-temperature 3D printing technology realized efficient construction of complicated structures with undersized and exquisite dimensions via multiple strut members.

### 3.4. Function Properties of Low-Temperature 3D Printing Technology

#### 3.4.1. Structure Strength of Low-Temperature 3D Printing PVA Matrix Hydrogels

Mechanical strength was the application base of low-temperature 3D printing PVA matrix hydrogels. Even though Figure 3 and Figure 4 exhibit perfect low-temperature 3D printing of simple and complex structures, the path structures were obtained from the slicing software of CuraEngine directly. The generated 3D printing paths only exhibited the original shape patterns, which ignored the effect of the parameters, including optimal path, angles, filling rate and layers, on mechanical strength. Therefore, design of a low-temperature 3D printing path and construction relationship between path structure and mechanical strength became the key points for mechanical property enhancement of low-temperature 3D printing PVA matrix hydrogels.

Because of the bottom-up conduction pattern, the top parts of the structure with high height had a relative low solidification property. The key point for successful low-temperature 3D printing was the cooperation of the extrusion speed, movement speed of the nozzle and freeze speed of the PVA matrix hydrogel reaction liquid. The arrangement of ancillary paths with relatively low 3D printing speed can provide enough time for sufficient solidification of the 3D printed PVA matrix hydrogel and realize 3D printing precision. The self-compiling G-codes, with all 3D printing parameters, built the programmable structure base. Figure 5a shows the designed path pattern of the parallel path structure. The dimensions of the parallel path structure with 10 layers and 100% filling rate were 40 mm × 5 mm × 3 mm (Length × Width × Height). The path structure was designed as a zigzag pattern. In order to realize an accurate intact dimension pattern, ancillary paths shown in Figure 5b were prepared via relatively low nozzle movement speed. After removing the ancillary paths, the finial low-temperature 3D printed PVA-LS hydrogel with parallel path structure is shown in Figure 5c. The supplemental movie (Supporting Information M9) exhibits the detailed low-temperature 3D printing process of the parallel path structure. In the first layer, the ancillary path was 3D printed in the periphery of the parallel path structure, as shown in Figure 5d. When the ancillary path was low-temperature 3D printed, the movement speed of nozzle was slow, which provided sufficient time for solidification of the parallel path structure. The existence of ancillary paths ensured the positive role of low-temperature for the construction of the parallel path structure layer by layer as shown in Figure 5e,f. The low-temperature 3D printing process proved the feasibility of self-compiling G-codes in low-temperature 3D printing technology.

Self-compiling G-codes enriched the designability of low-temperature 3D printing of the PVA matrix hydrogels. Inspired by the microstructure characteristic of bionic models with perfect mechanical strength, the bionic structures provide a perfect structure model for the design of the path structure with high mechanical strength via self-compiling G-codes. In our previous study, the mechanical properties of the spearer propodus of mantis shrimp was investigated [28]. Figure 6a exhibits the typical layered spiral structure of the spearer propodus of mantis shrimp. The spearer propodus (red wireframe in Figure 6a) bore a maximum tensile force of 320 N via an in situ tensile test [28]. Inspired by the layered spiral structures shown in Figure 6a, a series of layered spiral path structures with different layer angles were designed. Figure 6b exhibits the layered spiral path structure with dimensions of 40 mm × 5 mm × 3 mm (Length × Width × Height) via self-compiling G-codes. Based on the steady operation of the low-temperature 3D printing machine, the path structure with seven layers as shown in Figure 6b was low-temperature 3D printed with PVA-CMC as shown in Figure 6c. The supplemental movie (Supporting Information M10) exhibits the detailed low-temperature 3D printing process of the layered spiral path structure. The direction along with width was treated as 0°, as shown in Figure 6d. PVA-CMC hydrogel reaction liquid was smoothly extruded and maintained the 3D printed path structure with a 100% filling rate. The path direction angle between the first layer and the second layer was 30° in the counterclockwise direction. As shown in Figure 6e,f, the path direction angle of the third layer and the sixth layer was 60° and 150°, respectively. Namely, the path direction angle between adjacent layers was 30° in the counterclockwise direction, which was defined as a layered spiral path structure of 30°. Besides the path structure of 30°, the path direction angel of adjacent layers can also be designed as 15°, 45°, 60°, 75° and 90°. The PVA-CMC hydrogel with a layered variable-angle structure further verified the structural diversity of low-temperature 3D printing technology.

In order to verify the positive effect of layered variable-angle structures on the enhancement of mechanical strength, mechanical strength values of the low-temperature 3D printed PVA-CMC hydrogels with various path direction angels were investigated as shown in Figure 7a. The stress values of the PVA-CMC hydrogels with 0°, 15°, 30°, 45°, 60°, 75° and 90° were 153.1 KPa, 193.5 KPa, 398.8 KPa, 333.1 KPa, 235.5 KPa, 210.1 KPa and 207.1 KPa, respectively. The strain values of the PVA-CMC hydrogels with 0°, 15°, 30°, 45°, 60°, 75° and 90° were 65.7%, 68.8%, 101.4%, 106.5%, 77.6%, 74.2% and 72.3%, respectively. Compared with PVA-CMC hydrogels with 0°, PVA-CMC hydrogels with a layered variable-angle structure exhibited high mechanical strength. Moreover, PVA-CMC hydrogels with 30° had the highest stress values. Besides the PVA-CMC hydrogels, the low-temperature 3D printed PVA-LS hydrogels with a layered variable-angle structure also exhibited a high mechanical strength value of 372.2 KPa as shown in Figure 7b, which proved the mechanical strength enhancement of a layered variable-angle structure. Compared with our previous study [27], the low-temperature 3D printed PVA matrix hydrogels with a strength enhanced structure had the perfect mechanical property and application bases.

Based on Figure 5, low-temperature 3D printing technology maintained the original excellent mechanical properties of PVA matrix hydrogels via a self-compiling 3D printing path structure design. Moreover, low-temperature 3D printing technology realized diversified structure compiling properties in PVA matrix hydrogels with high structural strength.

#### 3.4.2. Conductivity and Sensing of Low-Temperature 3D Printing PVA Matrix Hydrogels

Low-temperature 3D printing technology realized high mechanical strength in PVA matrix hydrogels via the construction of a path structure. During the implementation process of the low-temperature 3D printing of PVA matrix hydrogels, 3D printable rheology of PVA hydrogel reaction liquids were controlled by low-temperature without any rheology modifiers, which maintained the original material components of the PVA matrix hydrogel. Namely, the original functional properties including electrical sensing characteristics can also be maintained logically. In order to verify the conductivity and sensing properties, novel self-made surface reduced silver reactions [27] were conducted on the low-temperature 3D printed PVA matrix hydrogels.

Figure 8a shows a low-temperature 3D printed PVA-LS hydrogel with a layered variable-angle structure of 30° after a reduction reaction of silver particles. Compared with Figure 5c, the colour of the PVA-LS hydrogel with Ag became dark and grey. The path structure patterns can still be observed. After immersing in the mixed AgNO_3_/PVP solution and the VC/PVP solution, silver particles were obtained on the surface of the PVA-LS hydrogels, as shown in Figure 8b. The energy spectrum analysis shown in Figure 8c disclosed the existence and distribution position of the silver particles. XRD analysis as shown in Figure 8d exhibited that the phase component of reduced silver on PVA-LS surfaces was an elementary substance. The novel self-made surface reduced silver reactions had steady and efficient characteristics, leading to the uniform size of silver particles as shown in Figure 8e,f. Based on the existence of silver particles, all surfaces of PVA-LS hydrogels had electrical conductivity, which can lighten “JLU” lighting sets with a low electrical resistance value as shown in Figure 8g (Supporting Information M11). Combined with the conductivity and low-temperature 3D printed soft structure with high mechanical strength, the PVA-LS hydrogel with a layered variable-angle structure of 30° realized 100 times the cyclic stretching processes with 5% strain. During the cyclic stretching process shown in Figure 8h, the output signal of ΔR/R_0_ was constant with minor fluctuations, indicating the stable output electrical signal changes under small strain of the low-temperature 3D printing conductive PVA-LS hydrogels.

**Figure 9:**
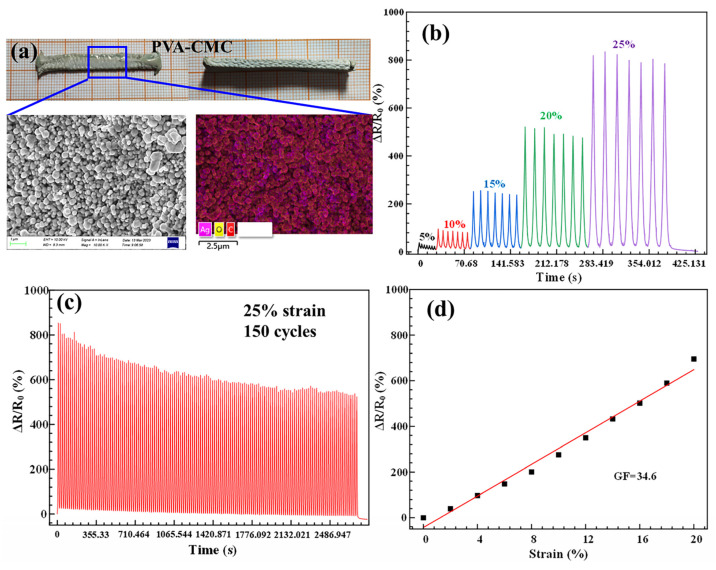
(**a**) material object and amplifying morphology of Ag particles, (**b**) ΔR/R_0_ with cyclic different strains, (**c**) ΔR/R_0_ with 25% strain for 150 cycles and (**d**) line-fitting curve of ΔR/R_0_ with continuous strain variation of low-temperature 3D printing PVA-CMC conductive hydrogels.

Besides PVA-LS hydrogels, the low-temperature 3D printed PVA-CMC hydrogels can also have electrical sensing functions via the reduction reaction of silver particles. Compared with pure PVA-CMC hydrogels as shown in Figure 6, the PVA-CMC hydrogels with silver particles also exhibited a dark and grey colour and maintained the original layered variable-angle structure. Based on the silver particles with uniform size shown in Figure 9a, the conductive PVA-CMC hydrogel also had a low electrical resistance value. As shown in Figure 9b, the low-temperature 3D printed conductive PVA-CMC hydrogel was stretched to 5%, 10%, 15%, 20% and 25% strain values constantly. The corresponding ΔR/R_0_ values were 0.377%, 0.96%, 2.59%, 5.23% and 8.35%, respectively. The output signal was stable and repeatable in the seven stretching-releasing cycle processes with corresponding strains, indicating the perfect sensing property within 25% strain. The low-temperature 3D printed conductive PVA-CMC hydrogel was continuously stretched with a maximum effective strain value of 25% and released for 150 cycles as shown in Figure 9c. This steady and durable sensing function exhibited the application feasibility of the low-temperature 3D printing technology. ΔR/R_0_ values of the low-temperature 3D printed conductive PVA-CMC hydrogel were measured via the increasing strain values to analyse sensing sensitivity as shown in Figure 9d. After linear fitting, the slope of the fitting equation was 34.6 in the 0–20% strain range. Moreover, the fitting equation had relatively high linearity (R^2^ = 0.98). The low-temperature 3D printed conductive PVA-CMC hydrogel exhibited high sensitivity, which was suitable for detecting small deformations as a strain sensor.

Based on the electrical sensing functions of the low-temperature 3D printed PVA matrix conductive hydrogels, the low-temperature 3D printing technology maintained the original material properties, realizing conductivity and sensing properties via an in situ reduction reaction of silver particles on PVA matrix hydrogel surfaces. Compared with the traditional moulding method, the low-temperature 3D printing technology had diversified design patterns of 3D printing path structures and original material characteristics of the PVA matrix hydrogel reaction liquids, which prepared new kinds of PVA matrix hydrogels with perfect mechanical strength and electrical sensing functions. The low-temperature 3D printing machine and technology provided new efficient methods for resolving bottlenecks of PVA matrix hydrogels and built a firm technology base for practical application in soft electrical sensing fields of PVA matrix hydrogels.

## 4. Conclusions

In order to realize functionalization of various 3D printing path structures of PVA matrix hydrogels under the premise of maintaining the original material function attributes, a self-made-innovative-low-temperature 3D printing machine and corresponding technologies were developed. By constructing various structure patterns and conducting functionalization analyses, the feasibility and effectiveness of the low-temperature technology was proved. The main conclusions are listed as follows:

(1) PVA matrix hydrogel reaction solutions exhibited specific low-temperature rheology properties. During the temperature declining process from 25 °C to −20 °C, viscosity values of the PVA matrix hydrogel reaction liquids increased gradually, exhibited the ability to be 3D printed and adequately proved the theoretical feasibility of the low-temperature 3D printing technology;

(2) The low-temperature 3D printing machine was composed of a hydraulic extrusion mechanism, a parallel-shaft drive mechanism, a low-temperature control device and an electric control device, which realized 3D printing of PVA matrix hydrogels in a low-temperature environment. When PVA matrix hydrogel reaction liquid was extruded at a specific position, the controllable and steady low-temperature environment provided appropriate low-temperature rheology characteristics for the construction of diverse structural patterns;

(3) The low-temperature 3D printing machine provided the mechanical equipment base for the preparation of PVA matrix hydrogels with different structures. Based on a layer-by-layer construction model, the simple structural patterns, including a cuboid structure, a torus structure and a quadrangular platform structure, were obtained. Based on the subsidiarity role of strut members, complex structural patterns with multiple thru holes were also obtained. The low-temperature 3D printing machine effectively combined the smooth extrusion and low-temperature solidification of PVA matrix hydrogel reaction liquids, building a firm technology foundation for constructing diverse structural patterns;

(4) The low-temperature 3D printing technology had 3D printing path design capability via self-compiling G-codes. Inspired by the microstructure of the spearer propodus of mantis shrimp, which exhibit high mechanical strength, a layered variable-angle structure was added into low-temperature 3D printed PVA-LS and PVA-CMC hydrogels, realizing perfect mechanical strength. Moreover, PVA-CMC hydrogels with 30° had the highest stress values. Under the premise of diverse complex structural patterns, low-temperature 3D printing technology enhanced mechanical strength of PVA matrix hydrogels via the positive role of 3D printing path design;

(5) Besides high structural strength, the low-temperature 3D printing technology also maintained the original reduction reaction matrix role of PVA matrix hydrogels. Based on reduced silver particles with unform size, low-temperature 3D printing PVA matrix hydrogels exhibited excellent electrical sensing functions. The low-temperature 3D printed conductive PVA matrix hydrogels had a low electrical resistance value, steady and durable signal output capability and high sensitivity and linearity, providing new and efficient methods for solving PVA matrix hydrogel bottlenecks and the practical application of electrical sensing conductive hydrogels.

## Figures and Tables

**Table 1:** Main components of low-temperature 3D printing machine.

Name	Model	Location
Fish-eye rod end joint bearing	SA3T/K	Parallel-shaft drive mechanism
Carbon Fiber Rods	Diameter 5 mm	
M3 Fish-eye rod hanging platform	PLA, 3D printed	
Bolt	M3	
Pulley	Mounted with M8 linear bearings	
Steel Shaft	M8	
Synchronous wheel	2GT, 16 teeth, M5	
Passive Wheel	2GT, M3	
Synchronous belt	2GT, 5 mm	
Stepper motors	42BYGH40S	
Stepper motors	42BYGH60-401A	Hydraulic extrusion mechanism
Synchronous wheel	2GT, 20 teeth, M5	
Synchronous wheel	2GT, 60 teeth, M8	
Silica gel tube	M5	
LUER taper	M5	
Injector	20 mL	
Extruder support	PLA, 3D printed	
Cryogenic system main control board	Arduino MEGA2560 R3	Low-temperature control device
Temperature Sensor	DS18B20	
Semiconductor chilling plate	19,906, 12 V, 10 W	
Cooling Fan	9025	
Cooling Fan	4010	
Aluminum plate	200 mm × 200 mm	
3D printer main control board	MKS Gen-L V2.1	Electric control device
limit switch	KW8	

**Table 2:** Detailed parameters of the selected hybrid stepping motor.

Parameters	Values	Parameters	Values
Nominal voltage	3.7 V	Rated current	2.3 A
Location torque	≥2.6 N·cm	No-load torque	15 N·m
Rotational inertia	110 g·cm^2^	Stepping angle	1.8°
Resistance	1.6 Ω	Rated speed	300 r/min
Direction of rotation	Axial clockwise	Wiring harness	4
Phase number	2	Diameter of axle	8 mm
Gravity	470 g		

**Table 3:** Low-temperature 3D printing parameters.

Parameters	Units	Values
Inner diameter of nozzle	mm	0.6
Environment temperature	°C	−20
Feed rate	mm^3^/s	37.17
Extrusion rate	mm/s	10
Moving speed multiplier	-	80–100
3D printing rate	mm/s	7
Layer height	mm	0.3
3D printing path	-	Self design
Filling rate	-	100%

## Data Availability

Not applicable.

## Supplementary Materials

The following supporting information can be downloaded at: https://www.mdpi.com/article/10.3390/s23198063/s1, M1. 3D printing process of PVA-LS hydrogel reaction liquid at 25 °C. M2. The operation process of low-temperature 3D printing machine. M3. The low-temperature 3D printing process of cuboid structure. M4. The low-temperature 3D printing process of torus structure. M5. The low-temperature 3D printing process of quadrangular platform structure. M6. The low-temperature 3D printing process of Model I. M7. The low-temperature 3D printing process of Model II. M8. The low-temperature 3D printing process of Model III. M9. The low-temperature 3D printing process of self-compiling G-codes of parallel path structure. M10 The low-temperature 3D printing process of self-compiling G-codes of layered spiral path structure. M11. The conductivity of low-temperature 3D printing conductive PVA-LS hydrogels.
