# Supplementary material for: Low-Temperature 3D Printing Technology of Poly (Vinyl Alcohol) Matrix Conductive Hydrogel Sensors with Diversified Path Structures and Good Electric Sensing Properties
Source: Sensors (Basel). 2023 Sep 24;23(19):8063. doi: 10.3390/s23198063 (PMC10575391; doi:10.3390/s23198063)
Supplement: Supplementary file 1 [file sensors-23-08063-s001.zip › Research highlights.pdf]

Highlights

1. The novel and practical low-temperature 3D printing technology was successfully developed.
2. Low-temperature 3D printing machine and technology realized various self-compiling complicated structures.
3. Low-temperature 3D printing PVA matrix hydrogels owned excellent structure strength and electrical sensing functions.
