# Supplementary material for: Low-Temperature 3D Printing Technology of Poly (Vinyl Alcohol) Matrix Conductive Hydrogel Sensors with Diversified Path Structures and Good Electric Sensing Properties
Source: Sensors (Basel). 2023 Sep 24;23(19):8063. doi: 10.3390/s23198063 (PMC10575391; doi:10.3390/s23198063)
Supplement: Supplementary file 1 [file sensors-23-08063-s001.zip › Supporting Information.pdf]

Supporting Information

Low-Temperature 3D Printing Technology of Poly (Vinyl Alcohol) Matrix Conductive Hydrogel Sensor with Diversified Path Structures and Good Electric Sensing Properties

Qian Zhao[1], Chang Liu[1], Yanjiao Chang*[2], Han Wu[1], Yihao Hou[1], Siyang Wu[1], Mingzhuo Guo[1]

1 The Key Laboratory of Bionic Engineering, Ministry of Education, Jilin University, Changchun 130025, China.

2 College of Food Science and Engineering, Jilin University, Changchun 130062, China.

M1. 3D printing process of PVA-LS hydrogel reaction liquid at 25 °C.

M2. The operation process of low-temperature 3D printing machine.

M3. The low-temperature 3D printing process of cuboid structure.

M4. The low-temperature 3D printing process of torus structure.

M5. The low-temperature 3D printing process of quadrangular platform structure.

M6. The low-temperature 3D printing process of Model I.

M7. The low-temperature 3D printing process of Model II.

M8. The low-temperature 3D printing process of Model III.

M9. The low-temperature 3D printing process of self-compiling G-codes of parallel path structure.

M10 The low-temperature 3D printing process of self-compiling G-codes of layered spiral path structure.

M11. The conductivity of low-temperature 3D printing conductive PVA-LS hydrogels.

Corresponding author. Tel/fax: +86 0431-82960086.
E-mail: changyj@jlu.edu.cn
